# Supplementary material for: Diagnostic Accuracy of an Offline CNN Framework Utilizing Multi-View Chest X-Rays for Screening 14 Co-Occurring Communicable and Non-Communicable Diseases
Source: Diagnostics (Basel). 2025 Dec 24;16(1):66. doi: 10.3390/diagnostics16010066 (PMC12785997; doi:10.3390/diagnostics16010066)
Supplement: Supplementary file 1 [file diagnostics-16-00066-s001.zip › diagnostics-3990233-supplementary.pdf]

**Section**	**Item**	**CLAIM Recommendation**	**Status in This Study**	**Manuscript Location**
**Title & Abstract**	1	Identify article as involving AI/ML	Yes-CNN-based AI clearly stated	Title, Abstract
	2	State the clinical role of the AI system	Yes-Screening, triage, decision support	Abstract, Introduction
**Study Design**	3	Describe study type (prospective/retrospective)	Yes-Retrospective	Methods
	4	Define clinical setting	Yes-ER, ICU, OPD, tertiary hospital	Methods
	5	Define intended population	Yes-Adult patients (≥18 years)	Methods
**Data**	6	Describe data sources	Yes-VinBig, NIH, and TUTH datasets	Methods
	7	Provide inclusion criteria	Yes-Clearly defined	Methods
	8	Provide exclusion criteria	Yes-Clearly defined	Methods
**Reference Standard**	9	Describe ground truth	Yes-3 radiologists + adjudication	Methods
	10	Describe annotation methods	Yes-Independent reads + consensus	Methods
**Data Handling**	11	Handling of missing data described	Yes- Images with poor quality excluded	Methods – Evaluation
	12	Data preprocessing described	Yes-Resizing, normalization	Methods – Preprocessing
	13	Data augmentation described	Yes-Albumentations, artifact simulation	Methods – Preprocessing
**Model Development**	14	Model architecture described	Yes-Custom CMRF-Net CNN	Methods – Model Architecture

	15	Training details reported	Yes-Epochs, batch size, optimizer, loss	Methods
	16	Hyperparameters reported	Yes-Fully reported	Methods
	17	Software frameworks disclosed	Yes-PyTorch, Albumentations, React/NodeJS	Methods
**Evaluation**	18	Test set defined	Yes-Hold-out test set (n=522)	Methods, Results
	19	Performance metrics defined	Yes-AUC, sensitivity, specificity, mAP	Methods
	20	Statistical uncertainty (CI) reported	No-Not performed	—
**Clinical Comparison**	21	Comparator described	Yes-Radiologist consensus	Methods
	22	Reader study reported	Yes-Silent clinical trial	Methods – Deployment
**Explainability**	23	Use of interpretability methods	Yes-Grad-CAM + color-coded boxes	Results
**Robustness**	24	Subgroup or robustness analysis	Yes-Tested on ICU/portable/poor-quality images	Results
**Error Analysis**	25	Error patterns analyzed	Yes-FP and FN patterns reported	Results – Error Analysis
**Generalizability**	26	External validation	No-Not performed	—
	27	Dataset shift discussed	Yes-LMIC vs public dataset shift discussed	Discussion
**Bias & Fairness**	28	Bias assessment	Yes-Class imbalance discussed	Discussion
**Deployment**	29	Clinical workflow integration described	Yes-PACS + offline deployment	Deployment

**Ethics**	30	IRB approval reported	Yes-IRB No: 25 (6-11)E2 082/083	Declarations
	31	Consent described	Yes-Waived (retrospective)	Declarations
**Transparency**	32	Funding disclosed	Yes-No external funding	Declarations
	33	Conflict of interest disclosed	Yes-None declared	Declarations
	34	Data availability statement	Yes-Available on request	Declarations
	35	Code availability statement	Yes- Available on request	Declarations
**Generative AI Use**	36	AI tool use reported	Yes-Grammarly disclosed	Declarations
